# Dialysis in the Elderly: A Practical Guide for the Clinician

**DOI:** 10.1155/ijne/9538115

**Published:** 2025-02-23

**Authors:** Aparna Satish, Jhalak Agrohi, Dharshan Rangaswamy, Ravindra Attur Prabhu, Shankar Prasad Nagaraju, Indu Ramachandra Rao, Mohan V. Bhojaraja, Srinivas Vinayak Shenoy

**Affiliations:** ^1^Department of Anaesthesia, Kasturba Medical College Manipal, Manipal Academy of Higher Education, Manipal 576104, Karnataka, India; ^2^Department of Nephrology, Kasturba Medical College Manipal, Manipal Academy of Higher Education, Manipal 576104, Karnataka, India

**Keywords:** conservative kidney management, dialysis in elderly, dialysis withdrawal, nutrition in elderly, vascular access

## Abstract

The increasing prevalence of elderly patients with end-stage kidney disease (ESKD) poses unique challenges in nephrology. These patients often present with multiple comorbidities, cognitive impairments, and frailty, which significantly impact treatment options and outcomes. Conservative kidney management (CKM) offers a viable alternative to dialysis for many elderly patients by focusing on symptom management and enhancing quality of life rather than merely prolonging life. However, clinicians face difficulties in approaching patients and deciding between CKM and dialysis. In addition, advocating for dialysis involves challenges in selecting the appropriate modality and vascular access. Nutritional management, often overlooked, is critical due to the high prevalence of protein-energy wasting and sarcopenia among elderly dialysis patients. Similar to the initiation of dialysis, there are dilemmas in determining when to withdraw from dialysis. This practical review aims to guide clinicians through the complex and challenging process of managing dialysis in the elderly, emphasizing a holistic, patient-centered approach that prioritizes quality of life. A multidisciplinary strategy, integrating clinical expertise and patient autonomy, is essential to address the complex needs of this vulnerable population.

## 1. Introduction

The global population is aging, leading to a rise in the incidence of elderly patients with both chronic kidney disease (CKD) and end-stage kidney disease (ESKD). According to the United States Renal Data System (USRDS) 2023, the incidence of ESKD is highest among those aged over 75 years (1581 cases per million) [1]. Similar trends are observed in European and Asian countries, where more elderly patients are reaching ESKD and being offered dialysis [2, 3]. Elderly patients with ESKD, especially those above 70 years, have significant comorbidities, cognitive dysfunction, and frailty, which influence outcomes [4–6]. They also have high rates of early mortality and frequent hospitalizations [7]. Problems associated with aging, such as frequent falls and cognitive decline, further complicate management [5]. The focus in the care of elderly ESKD patients has gradually shifted from hard clinical outcomes like mortality to more meaningful outcomes, such as maximizing quality of life. This shift includes comprehensive conservative care, where the goal is not necessarily to prolong longevity but to enhance the quality of life [8].

While there are tools available to help clinicians predict prognosis and plan treatment for their patients, many focus on survival outcomes rather than quality of life or functional status, which may be more relevant to elderly patients [9, 10]. In this review, we aim to simplify the process for clinicians making critical decisions for their elderly patients, particularly in predicting prognosis and deciding on renal replacement therapy. This includes choosing the type of dialysis or vascular access for those opting for hemodialysis (HD), as well as addressing nutritional aspects for elderly patients on dialysis. In addition, we discuss the challenging decisions involved in withdrawing from dialysis.

## 2. Decision Regarding Conservative Care Versus Dialysis

**Figure 1:**
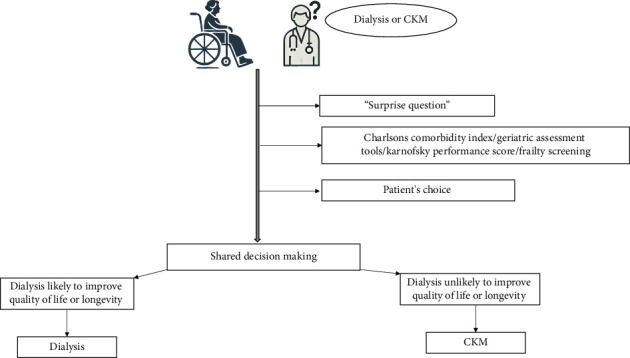
Approach to choosing between dialysis and conservative kidney management (CKM).

Conservative kidney management (CKM) or comprehensive conservative care is now part of the treatment options for elderly and highly comorbid ESKD patients. In many cases, CKM, which focuses on symptom management, quality of life, and avoiding the burdens of dialysis, can be a more appropriate option [11, 12]. Observational studies show no survival advantage for elderly patients with a high comorbidity burden who choose dialysis compared with CKM [4, 13–15]. In addition, dialysis may not improve survival and can result in greater hospitalizations, invasive procedures [16, 17], and functional decline [18]. Therefore, deciding the best course of action for elderly patients with advanced CKD involves a nuanced and patient-centered approach (Figure 1).

### 2.1. Patient Selection for CKM

Dialysis should never be the default treatment option for elderly patients reaching ESKD. Carefully selecting patients for CKM may ensure similar survival to dialysis but with a better quality of life [19, 20]. Most evidence for CKM comes from observational studies rather than randomized trials, making it challenging to strictly define criteria for choosing CKM over dialysis. Nevertheless, existing evidence can guide decision-making.

• “Surprise Question”: this tool relies on a nephrologist's intuition regarding the patient's overall health status. When asked, “Would you be surprised if this patient died in the next 12 months?” the nephrologist's response, either on a binary scale (yes/no) or a 5-point Likert scale (ranging from 1: definitely not surprised to 5: very surprised), helps prognosticate elderly ESKD patients. It has been shown to be associated with mortality [21, 22] and increased hospitalizations [23]. If the answer to the “surprise question” is “No” when considering treatment options for an elderly patient, the patient can be considered for CKM.
• Use of Validated Prognostic Tools/Scoring Systems/Prediction Algorithms: various instruments consider factors such as age, laboratory values, comorbidities, changes in comorbidity scores over time, functional status, and quality of life (QOL). These tools help predict outcomes in elderly patients [24–26]. In addition, geriatric assessment tools and assessments of frailty are used to decide on CKM [27–29]. Common geriatric screening tools encompass parameters such as chronological age, Karnofsky score, activities of daily living, instrumental activities of daily living, and self-reported health. Geriatric syndrome screening involves assessing walking speed, chair rise, frailty, cognitive function, falls risk, depression, and nutritional status [29]. These assessments contribute to a comprehensive understanding of patient wellbeing and guide clinical decision-making.
• Patient Choice: patient preference is essential in planning the management of elderly ESKD patients nearing dialysis. Studies have shown that many elderly patients, when fully informed about the potential outcomes and quality of life associated with dialysis, often opt for conservative management instead [30–32]. Patient preferences can be influenced by various factors, including cultural background, personal values, family opinions, and previous healthcare experiences. Individualized care plans that consider these factors are crucial. Some patients may prioritize longevity due to family responsibilities or personal goals, while others might emphasize comfort and palliative measures.

### 2.2. Shared Decision-Making-Integrating Clinical Evidence With Patient Autonomy

Shared decision-making is crucial for ensuring that choices align with the patient's values, goals, and preferences. This process involves open and honest communication where clinicians, patients, and their families collaborate to explore all treatment options, weighing potential benefits against burdens. A recommended framework from recent literature includes the following steps [33–35]:

• Initiate the discussion early and involve caregivers: start conversations about treatment options, including conservative management, well before dialysis becomes urgent. This allows time for patients and caregivers to understand their choices and avoid rushed decisions.
• Discuss prognostic information and address concerns: provide detailed and honest prognostic information, including potential survival rates, quality of life, symptom burden, and functional outcomes. Use simple language to avoid medical jargon. Encourage questions and address concerns during the discussion.
• Explore patient values and goals: engage in dialog to understand what matters most to the patient, such as longevity, quality of life, independence, and minimizing healthcare burden. This helps align treatment choices with their personal values and life goals.
• Review and reassess regularly: recognize that patient preferences and health status may change over time. Regularly revisit the discussion and reassess decisions to ensure they remain aligned with the patient's evolving situation and wishes.
• Document the decision-making process: keep detailed records of discussions, patient preferences, and agreed-upon plans. This documentation ensures continuity of care and can be referenced in future consultations.

## 3. Choosing the Dialysis Modality for the Patient

**Figure 2:**
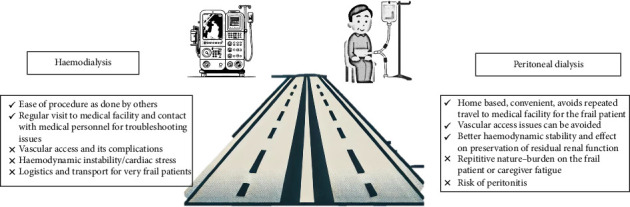
Advantages and disadvantages of hemodialysis and peritoneal dialysis in the elderly.

For elderly patients who decide to begin dialysis after careful consideration, choosing the appropriate modality is a challenge. The decision must consider the patient's characteristics, such as the high prevalence of comorbidities, cognitive dysfunction, frailty, and physical and functional dependencies. Observational studies and registry data provide mixed results regarding the benefits of one modality over another [36–39]. A large retrospective cohort study from Taiwan with propensity score matching showed no difference in all-cause mortality between HD and PD groups, but PD patients experienced a lower incidence of major cardiac and cerebrovascular events [40] (Figure 2).

### 3.1. HD for the Elderly: Advantages and Concerns

In-center HD offers substantial benefits for frail elderly patients compared to home-based peritoneal dialysis (PD). Key advantages include superior removal of waste products, less time required for treatment sessions, and reduced involvement needed from patients and their relatives in managing the dialysis process. In addition, attending a dialysis center allows elderly patients to engage with healthcare professionals and other patients, providing valuable social interactions that can be rare for isolated individuals [41]. However, concerns with HD for elderly patients include cardiovascular risks such as myocardial stunning, new-onset arrhythmias (including atrial fibrillation), and intradialytic hypotension, which can worsen cardiac status in frail patients [42–45]. In addition, the reliability of vascular access is an issue, as arteriovenous fistulas (AVFs) in the elderly are associated with higher rates of nonmaturation due to poor vascular caliber [46]. Consequently, central venous catheter use is higher in elderly patients, increasing the risk of infections and hospitalizations [47].

### 3.2. PD in the Elderly: Advantages and Concerns

PD offers several advantages for elderly patients, particularly those who are frail. It enables home-based treatment, fostering greater independence and flexibility compared to in-center HD [41]. PD integrates better with daily routines, reduces hospital visits, and avoids complications related to vascular access necessary for HD. In addition, PD helps maintain residual renal function longer, which is particularly beneficial for the elderly [48]. However, PD also has challenges. Elderly patients may find the physical demands of PD, such as handling dialysis bags and performing exchanges, difficult, especially if they have reduced manual dexterity or cognitive impairments. While the risk of peritonitis is not necessarily higher in older patients, it can have severe consequences if it occurs [49]. Assisted PD, where caregivers or healthcare workers aid with the procedure, can alleviate these issues but may not always be accessible or affordable [50].

### 3.3. Dialysis Targets in the Elderly and the Role of Incremental Dialysis

Traditional dialysis targets, such as achieving a specific Kt/V urea, may not be suitable for all elderly patients. Due to their often lower muscle mass, reduced physical activity, and higher prevalence of comorbidities, elderly patients may require adjusted targets that focus more on quality of life and symptom management rather than stringent biochemical goals [51]. For instance, a less aggressive fluid removal strategy can help avoid complications like intradialytic hypotension, common in older adults undergoing HD. Tailoring dialysis prescriptions to achieve minimal yet adequate solute clearance while preserving residual kidney function (RKF) is recommended for this population [52].

Incremental dialysis, where dialysis intensity is gradually increased as kidney function declines, is particularly beneficial for elderly patients. Starting HD with fewer sessions per week and gradually increasing the frequency can help maintain RKF and reduce cardiovascular events and mortality, as seen in a recent systematic review [53]. Similarly, incremental PD can start with fewer exchanges and increase based on the patient's residual renal function and clinical status. Studies have shown that incremental PD is associated with better preservation of RKF [54], lower peritoneal glucose exposure [55], and reduced rates of PD peritonitis [56, 57]. Incremental dialysis may also foster greater patient engagement and adherence by easing them into the treatment regimen and allowing more time for adjustment.

### 3.4. Effect of Dialysis on Cognitive Function in the Elderly

Elderly age is an important risk factor for the high prevalence of cognitive dysfunction in patients with ESRD. High prevalence of comorbidities such as hypertension, diabetes, prior cardiovascular events, and uremia-related toxicity make this population highly vulnerable to effects of cognitive decline [58, 59]. Cognitive dysfunction in elderly ESRD patients contributes to impairment in adherence to complex medical regimes, taking informed decisions and hampers their overall participation in health care management [60]. This leads to higher mortality, hospitalizations, and poor quality of life [61]. Hemodialysis is associated with ischemic injury to the brain on account of intradialytic hypotension(IDH) [62]. IDH leads to fluctuations in cerebral perfusion, causing subclinical ischemic injury that accelerates cognitive decline. Evidence suggests that these recurrent insults disproportionately affect attention, memory, and executive function [63]. In the absence of such hemodynamic instability, PD may theoretically offer better preservation of cognitive dysfunction but randomized studies in this regard are lacking. However, data from observational studies do suggest preservation of cognitive function in patients preferring PD over shorter periods of time [64, 65]. These findings underscore the importance of personalized dialysis modality selection and the need for further longitudinal studies to optimize cognitive outcomes in this vulnerable population.

## 4. Choosing the Vascular Access for the Elderly

**Figure 3:**
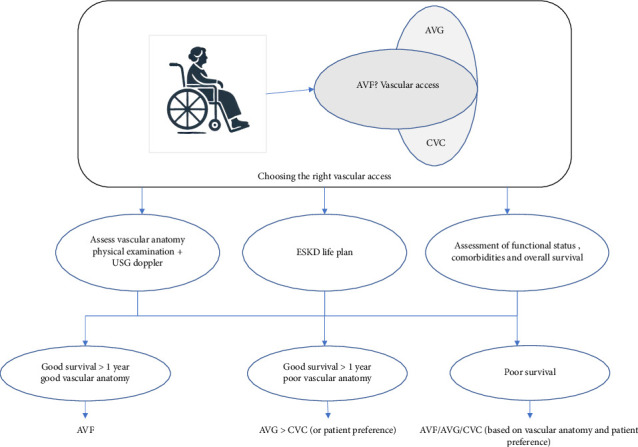
Choosing the right vascular access. AVF, arteriovenous fistula; AVG, arteriovenous graft; CVC, central venous catheter; ESKD, end stage kidney disease; USG, ultrasonography.

While planning vascular access for elderly patients for HD, a personalized approach focused on the patient's needs is crucial. Factors to consider include the vascular anatomy of the upper limbs, overall survival estimate considering comorbidities, anticipated time available before dialysis initiation, and patient preference (Figure 3).

### 4.1. Fistula First in the Elderly and the Concerns

Elderly patients often have poor vascular health characterized by atherosclerosis and diminished vessel elasticity, complicating the surgical creation and maturation of AV fistulas. The higher prevalence of comorbidities such as diabetes and hypertension further exacerbates these difficulties, leading to increased primary failure rates and prolonged maturation times for AV fistulas [66, 67]. While AV fistulas are traditionally preferred over AV grafts due to lower infection rates and longer patency, their initial failure rates and extended maturation periods pose significant drawbacks for older patients, leading to higher catheter dependence within the first 6 months [68]. Conversely, AV grafts offer quicker maturation and higher initial success rates, making them a viable alternative for elderly patients with poor vascular health.

### 4.2. Strategies to Ensure Appropriate Vascular Access

• Thorough presurgical mapping of upper limb vascular anatomy: comprehensive assessment through physical examination, ultrasound evaluation, or venography ensures optimal outcomes [69]. Poor vascular anatomy may preclude successful AVF creation, leading to consideration of AVG.
• Role of surgical expertise in fistula creation: fistulas created by experienced surgeons are associated with a 34% lower risk of early failure compared with those created by less experienced counterparts [70].
• Development of an “ESKD Life-Plan”: This has been advocated by the 2019 KDOQI guidelines [71]. Within the context of the ESKD Life-Plan, a vascular access plan must be individualized to the patient's specific needs. Developing such a plan involves considering several critical factors: the patient's anticipated life expectancy and prognosis, their baseline functional status, their personal preferences, the underlying vascular biology, the likelihood of successful AV fistula maturation or assisted maturation, the potential necessity for future maintenance interventions, and the impact of the vascular access choice on the quality of life for both the patient and their family [72].

### 4.3. Harnessing Newer Technology for Vascular Access in the Elderly

Recent advancements in endovascular techniques offer promising alternatives to traditional surgical methods, potentially reducing failure rates and improving patient outcomes [73–75]. Endovascular arteriovenous fistulas (endoAVFs) represent a significant innovation. Unlike traditional AVFs, endoAVFs like the Ellipsys Vascular Access System™ are created using minimally invasive endovascular procedures, reducing surgical trauma and associated complications, making them particularly advantageous for elderly patients with fragile vasculature and multiple comorbidities [76]. Like traditional surgically created AVFs, endoAVFs may be associated with worsening heart failure due to increased cardiac output. However, in the absence of long-term data and data from RCTs on the incidence of heart failure in patients undergoing endoAVFs, patient selection may hold the key to avoid such complications. Despite their promising results, endoAVFs may not yet be cost-effective in comparison to surgically created AVFs which is supported by a recent study from the United States (US) [77]. EndoAVFs are available only in few medical centers in North America and Europe which is an important limitation. Bioengineered blood vessels (BBVs) are emerging innovations having the potential to change the vascular access landscape. These vessels are created using tissue engineering techniques that involve culturing cells, often fibroblasts, onto biodegradable scaffolds, resulting in decellularized, biologically compatible vascular conduits [78]. A phase 2 trial in 3 centers in the US and Poland showed patency rates comparable with traditional synthetic grafts and a reduced risk of infection [79]. This is of particular interest in the elderly ESKD patients since BBVs are designed to overcome poor vascular quality. However, this will be significantly dependent on the cost barriers as well as long-term studies validating their efficacy and durability.

## 5. Addressing the Nutritional Aspects of the Elderly Dialysis Patient

### 5.1. Malnutrition in the Elderly

Malnutrition is highly prevalent among elderly CKD patients, especially those on dialysis [80]. The prevalence of protein-energy wasting (PEW) in this population ranges from 11% to 54%, increasing with the severity of kidney disease [81]. Sarcopenia, characterized by the loss of skeletal muscle mass and strength, affects up to one-third of elderly dialysis patients, significantly contributing to malnutrition [82]. The aging process, combined with metabolic derangements of CKD, exacerbates malnutrition risk, leading to poor clinical outcomes such as increased morbidity and mortality. Several mechanisms contribute to malnutrition in elderly CKD patients, including decreased dietary intake due to anorexia driven by uremia and altered taste perception, and systemic inflammation promoting muscle protein breakdown. Inflammatory markers like C-reactive protein (CRP) and interleukin-6 (IL-6) are frequently elevated in this population, correlating with higher rates of PEW [83–85]. Dialysis itself is a catabolic process that increases protein and energy requirements while causing nutrient losses through the dialysis membrane. Other contributing factors include metabolic acidosis, hormonal changes, and comorbidities such as diabetes and gastrointestinal disturbances [86, 87].

### 5.2. How to Screen and Manage Poor Nutritional Status

Effective screening for nutritional status in elderly CKD and ESKD patients is essential for early identification and management of malnutrition. The National Kidney Foundation Disease Outcomes Quality Initiative (KDOQI) recommends biannual nutritional assessments using tools such as the Subjective Global Assessment (SGA) and the Malnutrition Inflammation Score (MIS) [88]. These tools evaluate dietary intake, weight changes, functional status, and physical examination findings, providing a comprehensive assessment of nutritional health. Biochemical markers such as serum albumin and prealbumin, although useful, should not be solely relied upon due to their sensitivity to non-nutritional factors. Systemic inflammation, fluid overload, acute infections, altered renal clearances can affect the levels of albumin and prealbumin [89]. Hence, an integration of clinical assessment, dietary history and laboratory parameters are essential for a comprehensive approach to diagnose malnutrition in this population.

**Figure 4:**
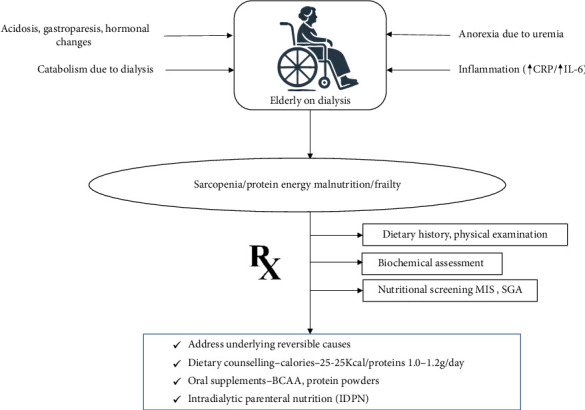
Pathophysiology, assessment and management of malnutrition in the elderly dialysis patient. CRP, C-reactive protein; IL-6, interleukin 6; MIS, malnutrition inflammation score; SGA, subjective global assessment; BCAA, branched chain amino acids.

Management of malnutrition in elderly CKD and ESKD patients involves a multifaceted approach focusing on adequate dietary intake, nutritional supplementation, and addressing underlying causes such as inflammation and metabolic acidosis. Recommended dietary protein intake for patients on dialysis is 1.0–1.2 g/kg/day, with energy intake recommendations of 25–35 kcal/kg/day [88]. Oral nutritional supplements (ONSs) in the form of branched-chain amino acids (BCAAs), protein powders with added calories, and unsaturated fat supplements have been tried in elderly dialysis patients, either as daily or intradialytic supplements [90–92]. Although albumin and prealbumin are poor markers of nutritional status when used in isolation because they are sensitive to non-nutritional factors, there is evidence that oral nutritional supplements improve these markers. These changes probably reflect broader positive shifts in nutritional and metabolic states, such as increased protein and energy intake, rather than providing an isolated measure of nutritional status. In addition, oral nutritional supplements have also been associated with increases in lean body mass, providing a more robust indication of their role in improving overall nutritional health in dialysis patients [93]. In cases where oral intake is insufficient, intradialytic parenteral nutrition (IDPN) can be considered, although its additional benefits over oral supplementation alone are limited [94, 95] (Figure 4).

## 6. When to Withdraw From Dialysis

**Figure 5:**
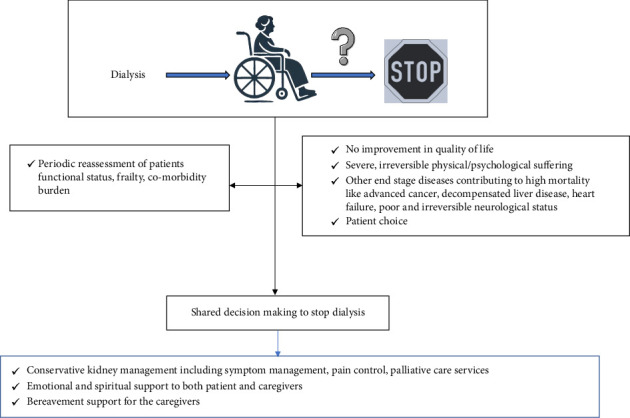
Approach to withdrawal from dialysis.

Withdrawal from dialysis is a critical and increasingly common decision in managing ESKD among the elderly. The prevalence of dialysis withdrawal varies significantly globally, influenced by regional practices and demographic characteristics. In developed countries, it is one of the leading causes of death in patients with ESKD, with data from registries suggesting nearly 30% prevalence [1, 96, 97]. The decision to withdraw typically correlates with several factors, including age, race, and underlying comorbidities, significantly impacting quality of life [98] (Figure 5).

### 6.1. Identifying Candidates for Dialysis Withdrawal

The Renal Physicians Association/American Society of Nephrology (RPA/ASN) and KDIGO guidelines support the decision to identify patients who may benefit from dialysis withdrawal, emphasizing thorough clinical assessments and ethical deliberations [99, 100]. Suitable patients may include the following:

• Those experiencing severe and irremediable physical or psychosocial suffering.
• Patients with decision-making capacity opting out of dialysis.
• Those with conditions where dialysis minimally prolongs life while extending suffering, especially in cases of coexistent malignancies, severe neurological disease or dementia, cardiac failure, or persistent vegetative state.
• Patients with a high risk of mortality through objective assessments of frailty, comorbidity burden, and functional status [101, 102].

### 6.2. Proceeding Toward Withdrawal and Postwithdrawal Care

Withdrawing from dialysis should involve a multidisciplinary team, including nephrologists, nurses, social workers, and potentially clergy, along with the patient and their family or caregivers. This team approach facilitates shared decision-making, ensuring all stakeholders understand the patient's values and healthcare goals [103, 104]. Effective communication and decision-making tools are essential to address both reversible and irreversible factors influencing the patient's decision. This framework supports ethical medical practice and enhances patient and family satisfaction with end-of-life care [105]. After deciding to withdraw dialysis, comprehensive palliative care is crucial to manage symptoms and maintain quality of life in the remaining days. Supportive care includes managing physiological changes such as fluid overload, pain, nausea, and sleep disturbances [106–108]. Emotional and spiritual care provided through counseling and family support ensures a dignified end of life [109]. Providing education on the natural progression of untreated ESKD, along with clear communication about end-of-life care options, constitutes a fundamental aspect of supportive care tailored for elderly patients.

## 7. Conclusion

The management of dialysis in elderly patients is multifaceted, requiring a nuanced approach that goes beyond traditional survival metrics. As the global population continues to age, the prevalence of elderly patients with ESKD is expected to rise, necessitating a multidisciplinary approach to care. The high comorbidity burden, cognitive dysfunction, and frailty observed in this demographic underscore the need for personalized treatment plans that prioritize quality of life over mere longevity. Future research and clinical practice should focus on several key areas to improve the care of elderly patients with ESKD.

• Development of age-specific tailored guidelines considering the unique physiological and psychological needs of elderly patients to standardize care and improve outcomes.
• Enhanced predictive tools that not only predict survival but also quality of life and functional status, integrating factors such as frailty, comorbidities, and patient preferences.
• Adoption of innovative technologies such as telemedicine and remote monitoring to enhance the management of elderly patients on dialysis, particularly those on home-based therapies like PD.
• Strengthening the role of multidisciplinary teams, including nephrologists, dietitians, social workers, and palliative care specialists, ensuring a holistic approach to patient care.
• Educational programs aimed at patients and their caregivers to empower them to make informed decisions and manage their care more effectively.

In conclusion, managing dialysis in the elderly requires a shift from traditional survival-focused approaches to more holistic, patient-centered care. By embracing a multidisciplinary, personalized approach and focusing on quality of life, we can improve the outcomes and wellbeing of this vulnerable population.

## Data Availability

This study is a review article that synthesizes findings from previously published literature. All data supporting this study are available from the original sources cited in the reference list.
